# Antigen-driven T cell-macrophage interactions mediate the interface between innate and adaptive immunity in histidyl-tRNA synthetase-induced myositis

**DOI:** 10.3389/fimmu.2023.1238221

**Published:** 2023-09-22

**Authors:** Daniel P. Reay, Tracy Tabib, Ying Wang, Timothy B. Oriss, Nicholas A. Young, Robert A. Lafyatis, Wael N. Jarjour, Paula R. Clemens, Dana P. Ascherman

**Affiliations:** ^1^Department of Medicine, University of Pittsburgh School of Medicine, Pittsburgh, PA, United States; ^2^Department of Medicine, University of Miami School of Medicine, Miami, FL, United States; ^3^Department of Medicine, Ohio State University School of Medicine, Columbus, OH, United States; ^4^Department of Neurology, University of Pittsburgh School of Medicine, Pittsburgh, PA, United States

**Keywords:** myositis, HRS (histidyl-tRNA synthetase), T cell, macrophage, innate immunity, adaptive immunity

## Abstract

**Introduction:**

Previous work in humans has demonstrated that both innate and adaptive immune signaling pathways contribute to the pathogenesis of idiopathic inflammatory myopathy (IIM), a systemic autoimmune disease targeting muscle as well as extra-muscular organs. To better define interactive signaling networks in IIM, we characterized the cellular phenotype and transcriptomic profiles of muscle-infiltrating cells in our established murine model of histidyl-tRNA synthetase (HRS)-induced myositis.

**Methods:**

Myositis was induced in wild type (WT) and various congenic/mutant strains of C57BL/6 mice through intramuscular immunization with recombinant HRS. Histopathological, immunohistochemical, flow cytometric, and transcriptomic assessments were used to characterize the functional relationship between muscle-infiltrating cell populations in these strains lacking different components of innate and/or adaptive immune signaling.

**Results:**

RAG1 KO mice developed markedly reduced muscle inflammation relative to WT mice, demonstrating a key requirement for T cells in driving HRS-induced myositis. While the reduction of mononuclear cell infiltrates in CD4-Cre.MyD88fl/fl conditional knockout mice and OT-II TCR transgenic mice highlighted roles for both innate and TCR-mediated/adaptive immune signaling in T cells, diminished inflammation in Lyz2-Cre.MyD88fl/fl conditional knockout mice underscored the importance of macrophage/myeloid cell populations in supporting T cell infiltration. Single cell RNA sequencing-based clustering of muscle-infiltrating subpopulations and associated pathway analyses showed that perturbations of T cell signaling/function alter the distribution and phenotype of macrophages, fibroblasts, and other non-lymphoid cell populations contributing to HRS-induced myositis.

**Discussion:**

Overall, HRS-induced myositis reflects the complex interplay between multiple cell types that collectively drive a TH1-predominant, pro-inflammatory tissue phenotype requiring antigen-mediated activation of both MyD88- and TCR-dependent T cell signaling pathways.

## Introduction

Idiopathic inflammatory myopathy (IIM) encompasses a group of autoimmune disorders in which muscle as well as extra-muscular tissues are inappropriately targeted for immune-mediated damage/destruction (1–3). Subsets of IIM are characterized by specific autoantibodies and distinctive clinical features that predictably involve different combinations of muscle, skin, joint, and lung pathology. Collectively, this burden of muscular and extra-muscular organ involvement contributes substantially to the morbidity and mortality of IIM, often with devastating consequences. Unfortunately, therapeutic efforts have been hampered by the incompletely defined pathogenesis of these disorders. As a result, treatment has relied on the use of global immunosuppression with corticosteroids and other chemotherapeutic/biologic agents that carry significant risk of infection and other side effects.

Although IIMs are generally unified by immunologic targeting of muscle tissue, more detailed analysis of individual subsets reveals clinical, immunological, and histopathological variations that indicate nuanced differences in pathogenesis. While the presence of distinctive autoantibodies suggests an underlying antigen driven component to these disorders, there is very little evidence that autoantibodies directly mediate tissue pathology (with the exception of immune-mediated necrotizing myopathy (4)). Given that the histological hallmarks of polymyositis and dermatomyositis (the main subtypes of IIM) include the predominant influx of T cells in tissues such as muscle and lung (5, 6), the likelihood is that autoantibodies reflect underlying antigen-specific T cells which drive disease—a conclusion that is supported by *in vitro* as well as *ex vivo* studies of human cells/tissues (7–9).

Because histidyl-tRNA synthetase (HRS=Jo-1) is among the most common targets of antigen-specific B/T cells in human IIM, we previously established a model of the multi-system anti-synthetase syndrome by immunizing mice with recombinant HRS/CFA emulsions (10). These studies demonstrated that mice immunized with murine versions of HRS (~95% homologous to human HRS) developed several key features of the anti-synthetase syndrome, including myositis and lung inflammation. Equally important, this tissue phenotype correlated with species-specific immune responses consisting of class-switched anti-murine HRS autoantibodies and antigen-specific T cell activation (10).

While this model was the first to produce a systemic phenotype resembling human disease, non-specific inflammatory responses to CFA complicated assessment of underlying mechanisms surrounding the breach of immune tolerance and resulting adaptive autoimmune responses targeting HRS. At the same time, work by Howard et al. demonstrating that HRS possesses intrinsic chemokine properties capable of stimulating lymphocytes and immature dendritic cells via CCR5 suggested that this autoantigen might be capable of activating the innate immune system as an initial factor driving the disease process (11). We therefore developed an alternative model of HRS-induced myositis based on intramuscular immunization with recombinant murine HRS in the absence of CFA/IFA (12). In this model, immunized mice develop a robust myositis phenotype characterized by T cell-rich infiltrates in a perimyseal as well as endomyseal distribution resembling the unique histopathological features found in muscle biopsies of human patients with Jo-1 antibodies and the anti-synthetase syndrome (12). Although antigen-specific T cell proliferation and production of class-switched autoantibodies suggest a role for adaptive immunity in this model, the development of myositis is absolutely dependent on MyD88, with substantial (but overlapping) contributions from TLR2 and TLR4 signaling (13). Intriguingly, disease induction can be uncoupled from production of high titer anti-HRS autoantibodies—again pointing to the predominant role of innate and cell-mediated immunity in this model as well as its human disease counterpart.

Even with these advancements in model development and the overall histopathological approximation of HRS-induced myositis to human IIM, the underlying mechanisms of this disease process remain undefined. Key unanswered questions include the relationship between antigen-specific B/T cells and other cell types (such as macrophages and fibroblasts) contributing to innate immune responses. Comparative assessment of HRS-induced myositis in wild type mice and knockout strains lacking various components of adaptive or innate signaling pathways provides a unique opportunity to dissect these relationships that are relatively underappreciated, but highly relevant to human disease—particularly in the anti-synthetase syndrome where macrophages comprise a significant portion of muscle-infiltrating cells (14–16). In this work, we therefore employed a combination of approaches to demonstrate that full expression of myositis requires key interactions between antigen-specific T cells, macrophages, and fibroblasts which profoundly impact cellular as well as tissue phenotype. As such, this novel model of inflammatory myopathy directly demonstrates critical links between antigen-driven innate and adaptive immune signaling networks that likely underly a range of systemic autoimmune diseases in humans.

## Methods

### Recombinant antigen

As previously described, the immunodominant amino terminal fragment of murine HRS (mHRS) was produced as a maltose binding protein (MBP) fusion protein consisting of amino acids 1-151 of mHRS linked to the carboxy terminal end of MBP (MA/MBP) (10). Heretofore designated as recombinant HRS, this bacterially expressed fusion protein was purified with amylose resin per the manufacturer’s protocol (New England Biolabs, Ipswich, MA), assessed by SDS-PAGE, dialyzed against PBS, and filter sterilized prior to intramuscular immunization of mice.

### Mice

Founder mice utilized in this study were purchased from The Jackson Laboratory (Bar Harbor, Maine) and included the following strains: C57BL/6J (Cat #000664), B6.129S7-Rag1tm1Mom/J (RAG1 Knockout (KO), Cat # 002216), B6.Cg-Tg(TcraTcrb)425Cbn/J (OT-II, Cat # 004194), B6.129S2-Cd4tm1Mak/J (CD4 KO, Cat# 002663), B6.129S7-*Il1r1^tm1Imx^*/J (IL-1R KO, Cat# 003245), B6.129P2-Lyz2tm1(cre)Ifo/J (Lyz2-Cre, Cat # 004781), B6.Cg-Tg(Cd4-cre)1Cwi/BfluJ (CD4-Cre, Cat # 022071) and B6.129P2(SJL)-Myd88tm1Defr/J (MyD88*^Flox/Flox,^* Cat # 008888). While CD4-Cre.MyD88*^Flox/Flox^* conditional KO mice were generated by crossing CD4-Cre mice with MyD88*^Flox/Flox^* mice (resulting in Cre-mediated excision of MyD88 in CD4+ as well as CD8+ T-cells), Lyz2-Cre.MyD88*^Flox/Flox^* conditional KO mice were produced by crossing Lyz2-Cre mice with MyD88 *^Flox/Flox^* mice—resulting in excision of MyD88 in cells of myeloid lineage. For each strain, F1 WT/Cre-WT/FL mice were crossed to produce WT/Cre-FL/FL mice. Backcrossing of F1 WT/Cre-FL/FL mice to WT/WT-FL/FL mice yielded additional WT/Cre-FL/FL experimental mice. All mice were genotyped prior to breeding/experimental use. Mice were provided with food and water *ad libitum* and housed in specific pathogen free mouse facilities under IACUC-approved protocols (University of Pittsburgh, Pittsburgh, PA).

### Disease induction and tissue processing

One hundred (100) μL of affinity-purified, filter-sterilized recombinant HRS (3-5 mg/mL) was administered via bilateral, intramuscular hamstring injection (50 μl/side) of 8-10 week old female experimental mice (n=5 mice/group) to induce myositis. Mice were sacrificed 17-18 days post-immunization for collection of muscle tissue, serum, draining lymph nodes, and spleens. Isolated hamstrings were bisected, placed into cassettes, and fixed in 10% formalin prior to paraffin-embedding for sectioning and subsequent hematoxylin and eosin (H&E) staining as well as immunohistochemical characterization with antibodies against CD3 (Abcam, Cat # 16669), CD4 (Abcam, Cat # 183685), CD8 (Abcam, Cat # 251609), CD45 (Cell Signaling, Cat # 70257), and CD68 (Cell Signaling, Cat # 977785). Representative H&E-stained muscle sections from a minimum of two independent experiments* were scored for levels of inflammation on a scale of 0 to 3 based on the intensity and distribution of cellular infiltrates: 0 = no inflammation, 1 = minimal, 2 = moderate, and 3 = severe inflammation. All slides were assessed by two individuals blinded to study group, and severity scores were then averaged to assess levels of inflammation in immunized mice. Images were obtained with an Evos FL Auto microscope/camera system and processed without cropping or alteration of brightness/contrast. *Experiments with CD4KO and IL-1R KO mice only performed once.

### Adoptive transfer experiments

Donor spleens were harvested from 6-month-old female CD4-Cre.MyD88*^Flox/Flox^* experimental and MyD88*^Flox/Flox^* (Cre-) control mice. Following processing of spleens into single cell preparations, approximately 2x10^7^ donor splenocytes were administered to female recipient RAG1 KO mice in 200μL of sterile 1X PBS via tail vein injection (n=2 mice/group). Two days after adoptive transfer, RAG1 KO recipient mice were immunized intramuscularly with 100 μL of recombinant HRS protein (50 μL/hamstring). Eighteen (18) days post-immunization, mice were euthanized for processing and analysis of muscle tissue as previously outlined.

### Single cell isolation

Muscle tissue subjected to single cell isolation was harvested and stored on ice in serum-free Dulbecco’s Modified Eagle Medium (DMEM) immediately prior to processing. Biochemical and mechanical dissociation of hindlimb mouse tissues for isolation of muscle-infiltrating cells from recombinant HRS- versus PBS-immunized mice was performed using the Miltenyi Biotec Skeletal Muscle Dissociation Kit and a gentleMACS Octo-Dissociator (Miltenyi Biotec, Gaithersburg, Maryland). Following this dissociation step, single cell suspensions were sequentially filtered using 70 micron and 30 micron cell strainers to eliminate residual muscle fibers and washed with 1X PBS. Isolated cells were resuspended in appropriate volumes of PBS for cDNA library preparation and scRNAseq analysis or frozen in media containing 10% DMSO for future flow cytometry analysis.

### Flow cytometry

Single cell suspensions of muscle-infiltrating cell populations derived from different mouse strains were prepared as described and analyzed by multi-channel flow cytometry using an Aurora flow cytometer (Cytek). For multicolor staining of cell samples, equivalent numbers of cells from each study group were treated with eFluor fixable viability dye (ThermoFisher Scientific, Waltham Massachusetts) at a 1:1000 dilution and then incubated with CD16/CD32 Monoclonal Antibody Fc block (eBioscience, San Diego, California) prior to addition of an antibody cocktail containing the following antibodies in a volume of 100μL/cell preparation: CD45-PE-Cy5 (1:50), CD3-Alexa Fluor 488 (1:50), CD90.2-APC (1:50), CD4-Brilliant Violet 711 (1:20), CD19-Brilliant Violet 510 (1:20), F4/80-Brilliant Violet 605 (1:20), and CD161-Brilliant Violet 650 (1:20) (eBioscience, San Diego, California). Unstained cell samples were utilized as background controls. UltraComp eBeads Compensation Beads (ThermoFisher Scientific, Waltham, Massachusetts) were labeled with each individual antibody utilized in the full antibody cocktail for deconvolution of spectral emission overlap (unmixing). Unmixing protocols were performed and verified prior to multicolor data acquisition using SpectroFlo software. Data were then analyzed for characterization and relative quantification of muscle-infiltrating cell populations using FlowJo software (v10.8.1).

### Single cell RNA sequencing

Muscle-infiltrating cell populations isolated from pooled groups of 3-5 mice immunized with recombinant HRS versus PBS were processed into single cell preparations (as described above), counted, mixed with reverse transcription reagents, and loaded into the Chromium Controller per the manufacturer’s instructions for 5’v2 chemistry. Following cleanup and cDNA amplification, a gene expression library (corresponding to 4000 cells/experimental condition) was constructed from 50 ng of amplified cDNA. In parallel, 2 microliter aliquots of the amplified cDNA were combined with 10X Genomics’ mouse T Cell Mix and B Cell Mix to profile the TCRs and BCRs of muscle-infiltrating lymphocytes. Resulting libraries were sequenced on the NovaSeq 6000 platform and aligned to 10X Genomics’ mouse references (refdata-gex-mm10-2020-A for gene expression and refdata-cellranger-vdj-GRCm38-alts-ensembl-5.0.0 for mouse TCR and BCR clonotypes) using Cellranger-6.1.2. Secondary analysis was performed using R 4.2.1 with the Seurat package (v4.3.0). This approach yielded differentially expressed genes (DEGs) in specific cell populations clustered using the Seurat R toolkit. Gene Ontology algorithms were used for analysis of pathways induced by HRS immunization in designated cell types.

### NicheNet analysis

This computational algorithm (17) was used to infer ligands expressed by designated “sender” cell populations based on gene expression profiles of target (“receiver”) cells that included macrophages and fibroblasts. With the Seurat wrapper implementation program, a set of potential ligands was identified based on NicheNet’s reference ligand-receptor data sources by intersecting differentially expressed genes from the sender and receiver cell populations with corresponding ligands and receptors from the database. Predicted ligands were then ranked by Pearson correlation coefficients. Average ligand gene expression levels and the percentage of cells expressing genes encoding predicted ligands were calculated for subpopulations of interacting sender cells (T cells, macrophages, fibroblasts) using the Seurat R toolkit.

### Statistical considerations

Severity scores for muscle inflammation were compared using the Mann-Whitney *U*-test, with significance based on a two-tailed p value <0.05. Additional statistical approaches included those outlined in previous sections describing scRNAseq and NicheNet Analysis.

### Study approval

All animal experiments in this study were performed under protocols approved by the University of Pittsburgh Institutional Animal Care and Use Committee.

## Results

### T cells are required for HRS-induced myositis

**Figure 1:**
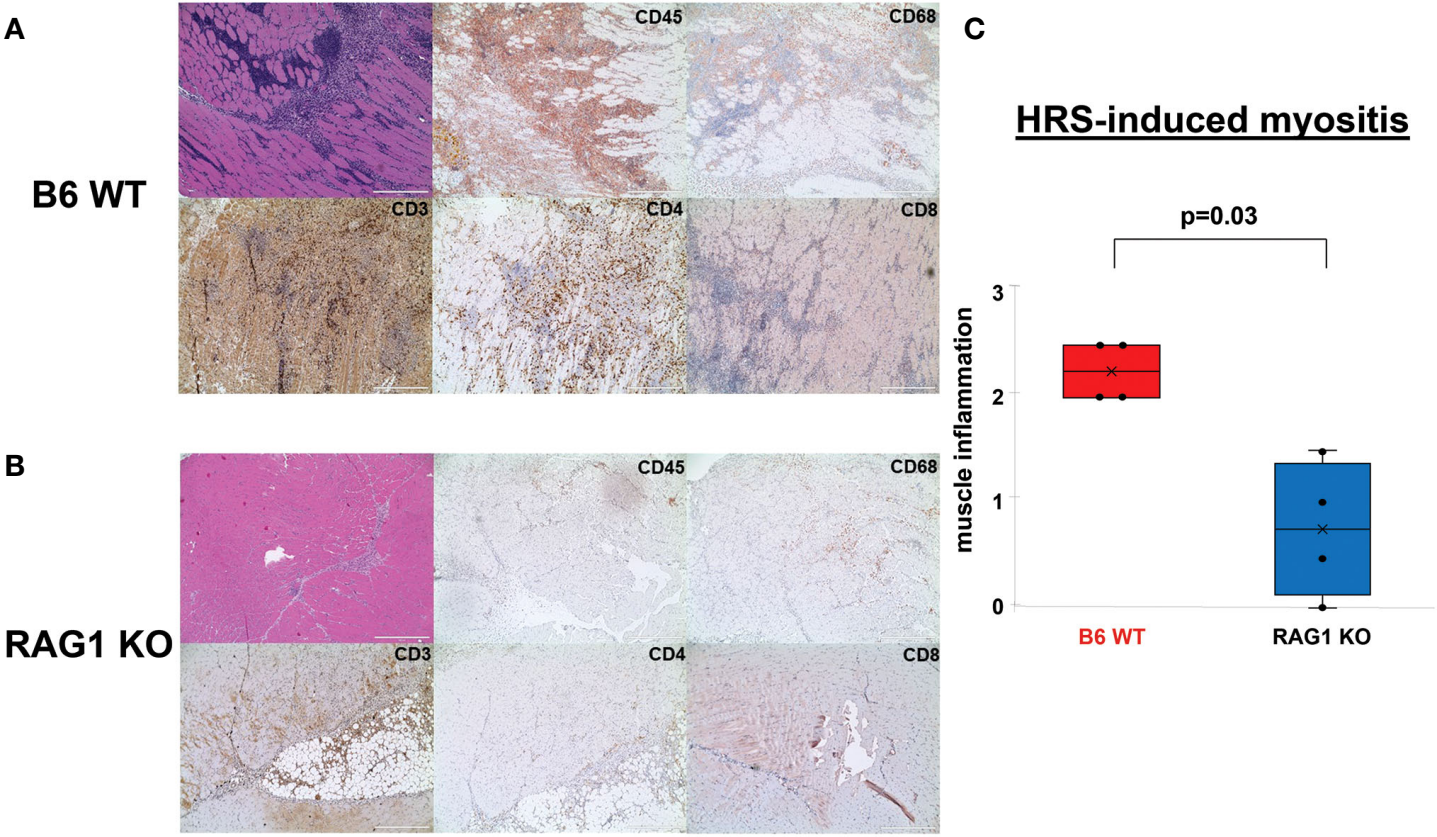
Role of T cells in HRS-induced myositis. **(A, B)** demonstrate H&E- and immunohistochemically-stained muscle tissue derived from n=4 C57BL/6 (B6) WT **(A)** and n=4 RAG1 KO **(B)** mice 17 days post immunization with recombinant HRS. Cell surface markers of infiltrating mononuclear cells (CD45), T cells (CD3, CD4, CD8), and macrophages (CD68) are indicated in the upper right hand corner of individual tissue sections. Scale bars (400 μm) are shown in the lower right hand corner of each photomicrograph. Box plots in panel **(C)** demonstrate the relative severity of muscle inflammation/cellular infiltration in B6 WT versus RAG1 KO mice following immunization with recombinant HRS. While horizontal bars designate median severity scores (median (IQR): 2.25 (2, 2.5) B6 WT vs. 0.75 (0.125, 1.375) Rag 1 KO), “x” symbols represent mean levels of inflammation (2.25 B6 WT vs. 0.75 RAG 1 KO). Minimum and maximum severity scores are indicated by whisker edges (min/max=2/2.5 B6 WT vs. 0/1.5 RAG1 KO); p-value determined by Mann-Whitney U-test.

To establish the importance of T cells in HRS-induced myositis, we immunized different strains of congenic C57BL/6 mice containing various defects in T cell distribution and/or signaling pathways with recombinant HRS, a fusion protein consisting of amino acids 1-151 of murine HRS fused to Maltose Binding Protein (MBP). As shown in **Figure 1**, C57BL/6 wild type [WT] mice immunized with recombinant HRS develop robust muscle inflammation in a mixed perivascular, endomyseal, and perimyseal distribution that peaks between Day 14-21 post immunization and is not seen in mice immunized with MBP alone (12) or MBP fused to the first 60 amino acids of murine HRS (13). Immunohistochemical staining and flow cytometric analysis of infiltrating cell populations reveals a mix of predominantly CD4^+^ T cells, B cells, NK cells, and macrophages (**Figure 1A** and **Supplementary Figure 1**). In contrast to WT mice, RAG1 KO mice demonstrate markedly reduced inflammation at equivalent time points (**Figure 1B**)—though there is clearly some infiltration of non-lymphocyte cell populations consisting primarily of macrophages (**Figure 1B** and **Supplementary Table 1**). Parallel immunization of CD4 KO mice yields an intermediate tissue phenotype, with clear reductions in the number of infiltrating CD3^+^ T cells and relative preservation of remaining subtypes of CD45^+^ cells that include CD68^+^ macrophages (**Supplementary Figure 2**).

### Innate immune signaling drives T cell infiltration in HRS-induced myositis

**Figure 2:**
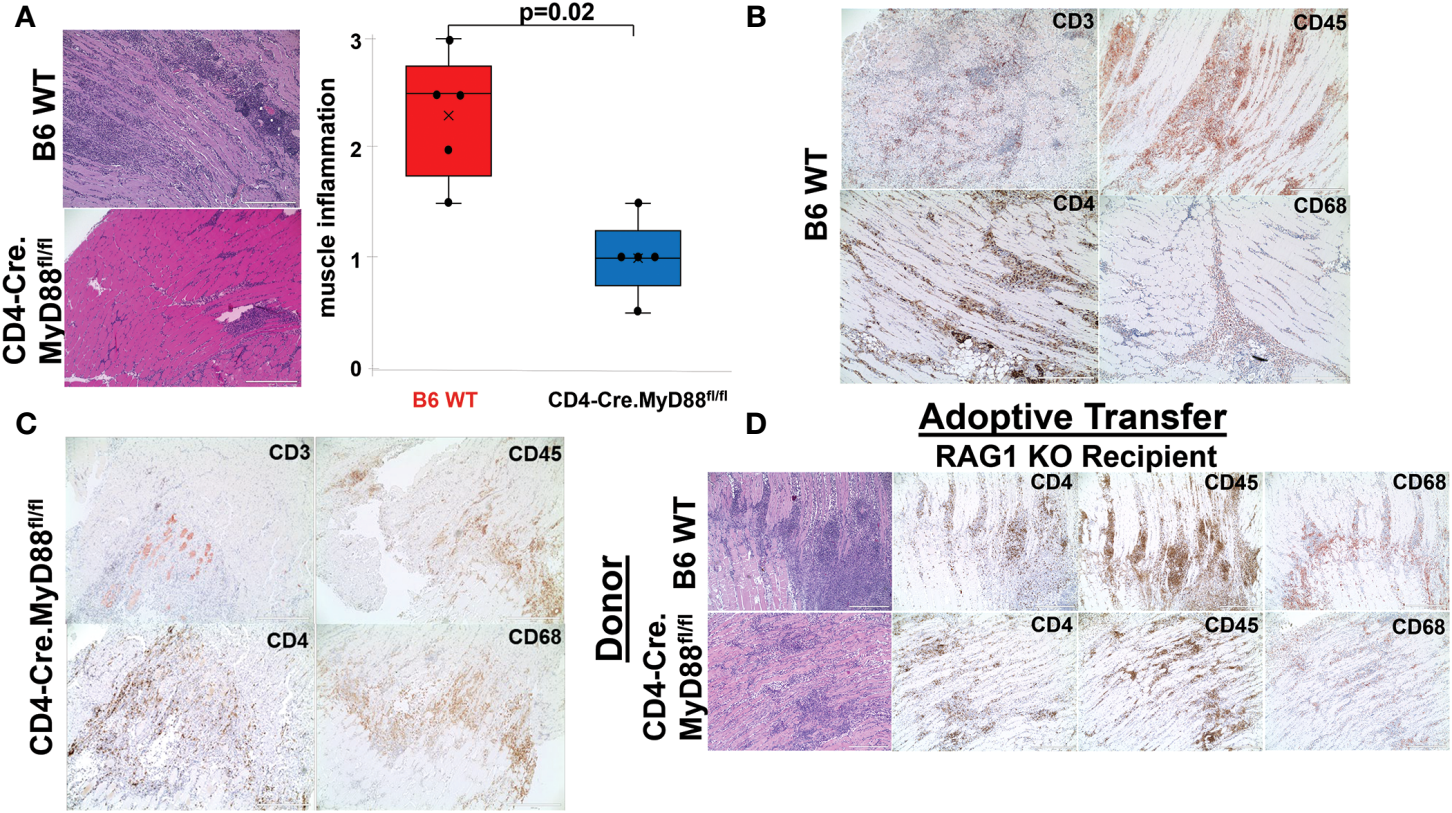
Requirement for T cell-specific MyD88 signaling in HRS-induced myositis. While the box plots in **(A)** demonstrate the relative severity of HRS-induced myositis in B6 WT versus CD4-Cre.MyD88^fl/fl^ conditional KO mice (n=5 mice/group) (median severity score (IQR)= 2.5 (1.75, 2.75) B6 WT vs. 1 (0.75, 1.25) CD4-Cre.MyD88^fl/fl^; min/max=1.5/3 B6 WT vs. 0.5/1.5 CD4-Cre.MyD88^fl/fl^; p-value determined by Mann-Whitney U-test), **(B, C)** depict immunohistochemical staining of muscle-infiltrating cell populations with antibodies recognizing CD45, CD3, CD4, and CD68. **(D)** demonstrates representative staining of muscle tissue derived from HRS-immunized RAG1 KO mice following adoptive transfer of unfractionated splenocyte populations from B6 WT versus CD4-Cre.MyD88^fl/fl^ conditional KO mice. Scale bars (400 μm) are shown in the lower right hand corner of each photomicrograph.

Based on the rapid time course of myositis development and the known dependence of this model on MyD88 signaling pathways, we sought to further define the role of innate immune responses in driving T cell infiltration following immunization with recombinant HRS (which we have previously shown can engage with endogenous/exogenous ligands to synergistically activate both TLR2 and TLR4 (13)). As a first step in examining the functional impact of T cell-intrinsic MyD88 signaling, we generated conditional KO mice lacking expression of MyD88 in CD4^+^ and CD8^+^ T cells by crossing C57BL/6 CD4-Cre founder mice with MyD88^fl/fl^ mice. Comparison of HRS-induced myositis in WT versus CD4-Cre.MyD88^fl/fl^ conditional KO mice demonstrates significant reduction in the severity of myositis (**Figure 2A**) that reflects diminished infiltration of both CD3^+^CD4^+^ T cells and CD3^-^CD4^-^CD45^+^ non-lymphoid cells that include CD68+ macrophages (**Figures 2B, C**; **Supplementary Figure 3**). Similarly, adoptive transfer of splenocytes from WT versus CD4-Cre.MyD88^fl/fl^ conditional KO mice into RAG1 KO mice demonstrates reduced recruitment of CD3^+^CD4^+^ T cells (as well as more global CD45^+^ cell populations) following recombinant HRS immunization of recipient mice reconstituted with CD4-Cre.MyD88^fl/fl^ donor lymphocytes (**Figure 2D**). While these results do not prove that HRS directly engages TLRs or other innate immune receptors expressed in muscle-infiltrating T cells, the development of florid myositis in HRS-immunized IL-1 Receptor KO mice (**Supplementary Figure 4**) suggests that phenotypic alterations in CD4-Cre.MyD88^fl/fl^ conditional KO mice are not simply driven by reduced MyD88-dependent responses to cytokines elaborated by other cell types such as macrophages or dendritic cells. Moreover, the proportionate reduction in subsets of CD3^-^CD4^-^CD45^+^ muscle-infiltrating cell populations observed in CD4-Cre.MyD88^fl/fl^ mice immunized with recombinant HRS (confirmed by flow cytometry and scRNAseq-based clustering, **Supplementary Figure 5** and **Supplementary Table 1**) indicates that influx of innate immune cell populations such as macrophages and NK cells is at least partially dependent on interactions with T cells activated through MyD88-dependent signaling cascades.

### Antigen-specific T cell signaling is required in HRS-induced myositis

**Figure 3:**
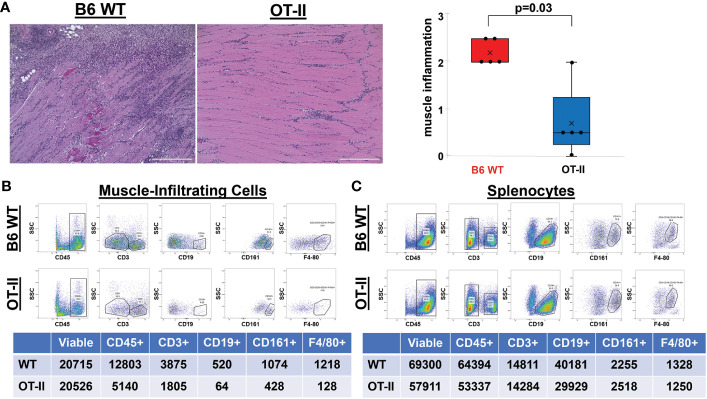
Contribution of antigen-specific TCR signaling to HRS-induced myositis. **(A)** demonstrates the relative severity of muscle inflammation in n=5 B6 WT versus n=5 OT-II (Ovalbumin-specific TCR transgenic) mice 17 days post-immunization with recombinant HRS. Scale bars (400 μm) are shown in the lower right hand corner of each photomicrograph. Box plots represent the interquartile range of muscle severity scores and include median (horizontal bars) as well as mean (“x” symbol) values; median (IQR)=2 (2, 2.5) B6 WT vs. 0.5 (0.25, 1.25) OT-II; mean=2.2 B6 WT vs. 0.7 OT-II; min/max=2/2.5 B6 WT vs. 0/2 OT-II; p-value determined by Mann-Whitney U-test. Dot plots and summary tables in panels B-C illustrate flow cytometric profiles of pooled muscle-infiltrating **(B)** and spleen **(C)** cell populations derived from n=4 B6 WT and n=3 OT II TCR transgenic mice following HRS-mediated induction of myositis. While CD19^+^ B cells were gated as a percentage of CD45^+^CD3^-^ populations, CD161^+^ NK cells and F4-80^+^ macrophages were quantified from CD3^-^CD19^-^ and CD3^-^CD19^-^CD161^-^ cells, respectively. Given the similar number of live cells isolated from muscle or spleen for each experimental condition, percentages of cell subsets relative to number of live cells have been omitted from tables for ease of illustration.

Given the residual T cell infiltration in CD4-Cre.MyD88^fl/fl^ conditional KO mice immunized with recombinant HRS, we next addressed the relative role of TCR-mediated, adaptive immune signaling pathways in driving HRS-induced myositis. To uncouple TCR-directed and innate immune receptor-mediated T cell activation, we employed OT-II transgenic mice that express an OVA-specific TCR and are incapable of engaging HRS in the context of peptide-MHC complexes. Histopathologic comparison of myositis severity in OT-II versus WT mice 17 days post recombinant HRS immunization revealed marked reduction in cellular infiltrates in OT-II mice (**Figure 3**), indicating that antigen-specific TCR-mediated signals are required to fully reproduce the phenotype of HRS-induced myositis. More detailed flow cytometric assessment of muscle-infiltrating cell populations demonstrated a global reduction in CD45^+^ subsets that impacted not only CD3^+^Thy1.2^+^CD4^+^ T cells, but also CD19^+^ B cells, CD161^+^ NK/NK T cells, and F4-80^+^ macrophages (**Figure 3B**)—again pointing to the importance of reciprocal interactions between T cells and other lymphoid as well as non-lymphoid cell populations (**Supplementary Table 1**). Of note, parallel flow cytometric analysis of splenocytes from HRS-immunized OT-II and WT mice showed equivalent representation/distribution of these cell types in both strains (**Figure 3C**), indicating that the observed differences in muscle-infiltrating cell populations were not due to baseline cellular depletion in either lymphoid or myeloid compartments of OT-II TCR transgenic mice. Beyond these considerations, demonstration of oligoclonal T cell expansion in WT mice through single cell TCR sequencing (**Table 1**) provided even more direct evidence that antigen-specific, TCR-mediated signals play a central role in HRS-induced myositis.

**Table 1:** T cell Clonotypes in HRS-induced Myositis*.

Clonotype	Number (%)^†^
TRB : CASGDGYSGNTLYF;TRA : CALGERSNNRLTL	9 (2.0)
TRB : CASGDWGGAETLYF;TRA : CAVSLMATGGNNKLTF	7 (1.6)
TRB : CASSSPTGGNQAPLF;TRA : CALSEGNNAGAKLTF	6 (1.3)
TRB : CASGGSGTGGYEQYF;TRA : CALSPSNMGYKLTF	5 (1.1)
TRB : CASRGGAGNTLYF;TRB : CASSVGQGAANERLFF;TRA : CALSMVSGTYQRF	5 (1.1)
TRB : CASSRTGSNERLFF;TRA : CAAVRGTGSKLSF	5 (1.1)
TRB : CASSSGQNNNQAPLF;TRA : CALDYNVLYF	5 (1.1)
TRB : CTCSADPGNRNTEVFF;TRA : CAADNTNTGKLTF	5 (1.1)
TRB : CASSDEIGVLYEQYF;TRB : CAWSLLGNYAEQFF;TRA : CALELANTGKLTF	4 (0.9)
TRB : CASSDSGTGVQAPLF;TRA : CAANANSGTYQRF;TRA : CAIEHNTNTGKLTF	4 (0.9)
TRB : CASSIVGTPGNTLYF;TRA : CAADTNTGKLTF	4 (0.9)
TRB : CASSLQAGWEQYF;TRB : CGARGGNTEVFF;TRA : CALGDGPSAGNKLTF	4 (0.9)
TRB : CASSPTGADTEVFF;TRA : CALSRSNYQLIW	4 (0.9)
TRB : CASTGRRNTGQLYF;TRA : CALSEWSNNRIFF	4 (0.9)
TRB : CAWSPGASAETLYF;TRA : CALGSNSAGNKLTF	4 (0.9)
IGH : CARGGWLAYW;IGK : CWQGTHFPWTF	3 (0.7)
TRB : CASGDAWGGAYEQYF;TRA : CVLRGNQGGSAKLIF	3 (0.7)
TRB : CASGDAWGSAETLYF;TRA : CAVRQGGNTGKLIF	3 (0.7)
TRB : CASGEDWGGNYAEQFF	3 (0.7)
TRB : CASGEGRSAETLYF;TRA : CAASATSSGQKLVF	3 (0.7)
TRB : CASSDSPYYEQYF;TRA : CAASGPNTGKLTF	3 (0.7)
TRB : CASSLGGGDFEQYF;TRA : CVLSNNNNAPRF	3 (0.7)
TRB : CASSLGQYEQYF;TRA : CALGYDTNYKVIF	3 (0.7)
TRB : CASSLLGGDAEQFF;TRA : CVLSNNNNAPRF	3 (0.7)
TRB : CASSLQLYF;TRB : CASSPGLGASAETLYF;TRA : CVLGRNSNNRIFF	3 (0.7)
TRB : CASSLTGGEEQYF;TRA : CAVRRNSNNRIFF	3 (0.7)
TRB : CASSPLTGVDTQYF;TRA : CAVGRYGSSGNKLIF	3 (0.7)
TRB : CASSQDGGAGLSQNTLYF;TRA : CAMRGNTEGADRLTF	3 (0.7)
TRB : CGARDPGHTGQLYF;TRA : CALSTSGGNYKPTF	3 (0.7)

*TCR CDR3 sequences.

**^†^** Number (%) of TCR clones with designated CDR3 sequences out of 447 total TCR clones.

### T cell signaling/activation drives transcriptional profiles and phenotype of macrophages in HRS-induced myositis

**Figure 4:**
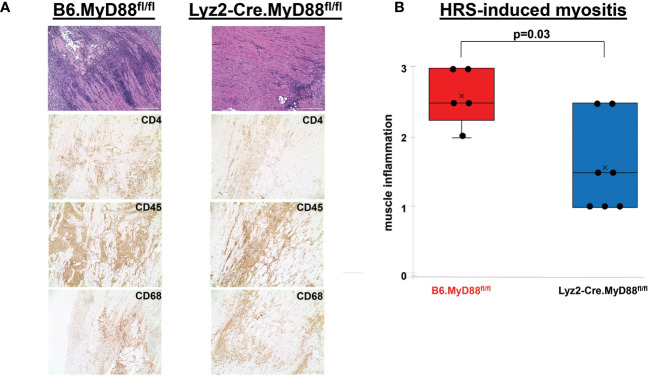
Dependence of HRS-induced myositis on myeloid/macrophage-specific MyD88 signaling. **(A)** depicts high (100x) power views of representative H&E-stained muscle tissue from Lyz2-Cre.MyD88^fl/fl^ conditional KO mice versus B6.MyD88^fl/fl^ (Cre^-^) littermate controls 17 days post immunization with recombinant HRS. Accompanying immunohistochemical stains demonstrate the distribution of CD4^+^ T cells, CD45^+^ leukocytes, and CD68^+^ macrophages in muscle-infiltrating cell populations. Scale bars (400 μm) are shown in the lower right hand corner of each photomicrograph. The box plots in **(B)** demonstrate the relative severity of muscle inflammation resulting from HRS immunization of n=5 B6.MyD88^fl/fl^ littermate control and n=7 Lyz2-Cre.MyD88^fl/fl^ conditional KO mice. While horizontal bars designate median severity scores, “x” symbols represent mean levels of inflammation; median (IQR)=2.5 (2.25, 3) B6.MyD88^fl/fl^ vs. 1.5 (1, 2.5) Lyz2-Cre.MyD88^fl/fl^; mean=2.6 B6.MyD88^fl/fl^ vs. 1.6 Lyz2-Cre.MyD88^fl/fl^; min/max=2/3 B6.MyD88^fl/fl^ vs. 1/2.5 Lyz2-Cre.MyD88^fl/fl^; p-value determined by Mann-Whitney U-test.

More detailed analysis of **Figure 3B** reveals a strikingly disproportionate reduction of muscle-infiltrating F4-80^+^ macrophages (relative to T cells) in HRS-immunized OT-II mice, with a macrophage: T cell ratio of 0.07 in OT-II mice versus 0.31 in WT mice. To define the requirement for myeloid cells such as macrophages and further assess their interaction with T cells in HRS-induced myositis, we generated myeloid-specific MyD88 conditional knockout mice in which Cre recombinase is expressed under the Lyz2 promoter (Lyz2-Cre.MyD88^fl/fl^ mice). As shown in **Figure 4**, these mice developed significantly less inflammation relative to WT/littermate control mice following immunization with recombinant HRS. Immunohistochemical staining demonstrates preferential reduction in CD4+ T cell infiltration of involved muscle tissue, indicating that myeloid cells with intact MyD88 signaling pathways are needed to fully recruit/activate muscle-infiltrating T cells (as well as B cells, NK cells, and other CD3^-^ cell types; **Supplementary Table 1**) through mechanisms that likely range from direct antigen presentation to elaboration of pro-inflammatory cytokines.

**Figure 5:**
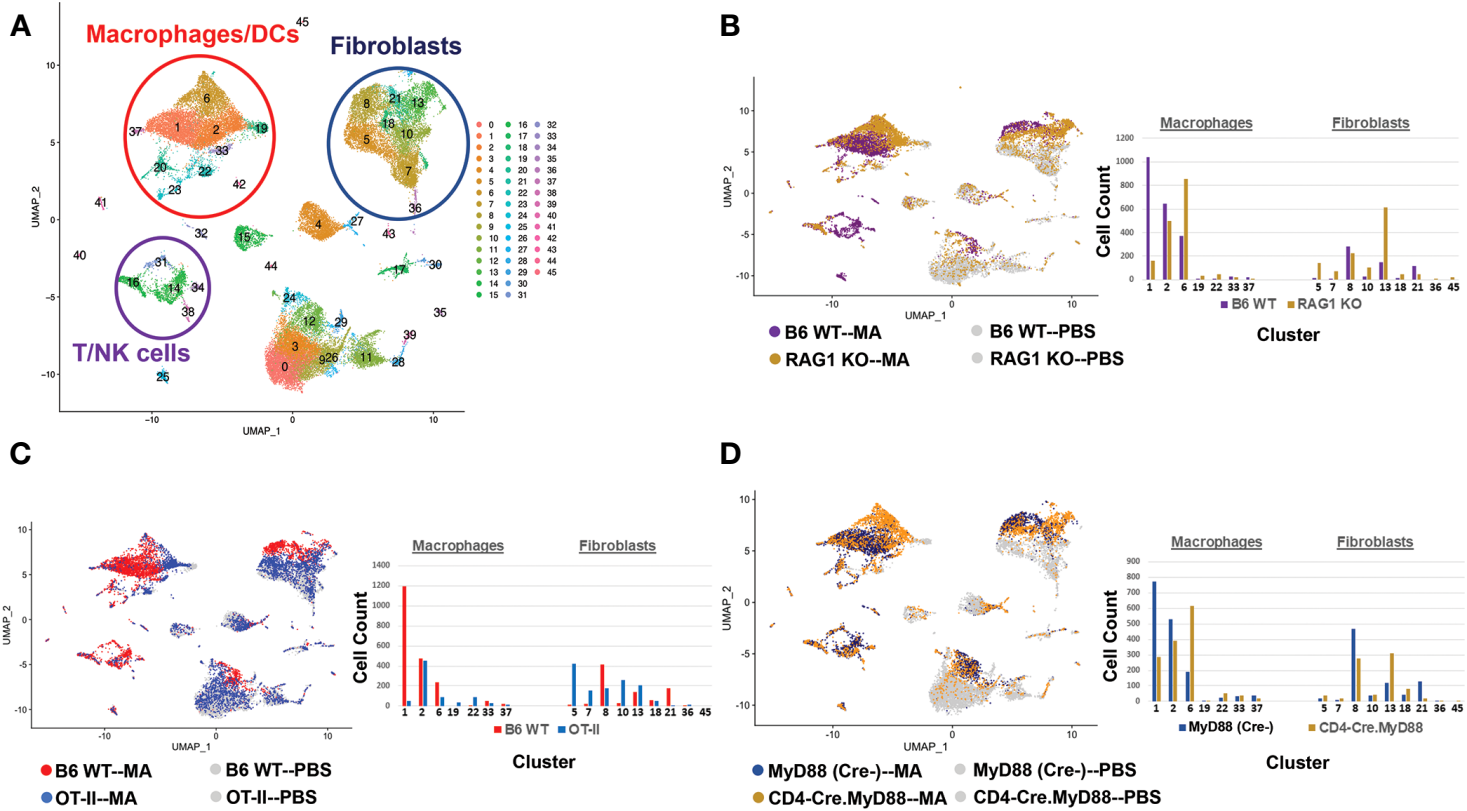
T cell activation signals and their impact on macrophage phenotype in HRS-induced myositis. The UMAP plot in **(A)** represents composite single cell RNA sequencing (scRNAseq)-based clustering of both infiltrating and resident cell populations isolated from muscle tissue of B6 WT mice following immunization with recombinant HRS versus PBS. The UMAP plots and accompanying bar graphs in **(B–D)** show the relative distribution of macrophage and fibroblast subsets following HRS-mediated induction of myositis in B6 WT versus RAG1 KO mice **(B)**, B6 WT versus OT-II TCR transgenic mice **(C)**, and CD4-Cre.MyD88^fl/fl^ conditional KO versus B6.MyD88^fl/fl^ (Cre^-^) littermate control mice **(D)**. Each of these comparative analyses represents scRNAseq profiling of muscle tissue from pools of n=3-5 mice; PBS controls have been omitted from the bar graphs of **(B–D)** due to limited populations of infiltrating macrophages/fibroblasts in these mice and overall ease of presentation. Additional clusters depicted in panel A include B cells/plasma cells (clusters 25/32), neutrophils (cluster 41), endothelial cells (clusters 0,3,9,12,24,26), and muscle satellite cells (cluster 4).

Coupled with the experiments depicted in **Figures 1**–**3**, the marked reduction of muscle inflammation in Lyz2-Cre.MyD88^fl/fl^ mice underscored key roles for both myeloid cells and T cells activated through innate and adaptive immune signaling pathways. We therefore performed comparative scRNAseq analysis of WT, OT-II TCR transgenic, CD4-Cre.MyD88^fl/fl,^ and RAG1 KO mice immunized with recombinant HRS to define the impact of targeted disruption of T cell activation/signaling on the relative distribution and transcriptomic profiles of different macrophage subsets in HRS-induced myositis. As shown in **Table 2**, HRS-mediated induction of myositis results in a dramatic shift from M2-like macrophages expressing CD209f/C209g/CD163/Lyve1 marker genes (cluster 19) to macrophages expressing genes associated with alternative phenotypes (clusters 1, 2, and 6). Review of **Figure 5** and **Table 3A**/**3B** clearly indicates that, in the absence of full T cell activation, macrophage populations are skewed towards subsets contained in clusters 2 and 6. In contrast, macrophages in cluster 1 are preferentially expanded in WT and MyD88^fl/fl^ (Cre negative) littermate control mice. Collectively, these data demonstrate that both innate and adaptive immune signals *in T cells* are needed for maximal induction of macrophage cluster 1, whereas MyD88-driven T cell signaling plays a more dominant role in modulating/limiting HRS-induced expansion of macrophage cluster 6 (compare relative induction of this cluster in RAG1 KO and CD4-Cre.MyD88^fl/fl^ conditional KO vs. OT-II TCR transgenic mice).

**Table 2:** HRS-induced Shifts in Macrophage Subsets of WT Mice.

Macrophage Cluster	PBS*	HRS^†^
1-Mcemp1 F10 Fpr1 Fpr2 Mac	11 (0.1)	3009 (23.8)
2-Marco Gbp2b Clec10a Mac	14 (0.1)	1650 (13.0)
6-Trem2 Spp1 Msr1 Acp5 Mac	6 (0.1)	802 (6.3)
19-Cd209f Cd209g Cd163 Lyve1 Mac	227 (2.4)	3 (<0.1)
22-Dcstamp Ear2 Retnla Cd226 Mac	128 (1.3)	45 (0.4)
33-Siglec1 Marco Mertk Mac	0 (0)	110 (0.9)
37-Treml4 Cd300e Ear2 Mac	18 (0.2)	80 (0.6)

*cell numbers (%) per cluster; total number of cells 9,532.

**^†^** cell numbers (%) per cluster; total number of cells 12,664.

**Table 3A:** T cell Interactions with Macrophages and Fibroblasts following Immunization with Recombinant HRS.

Cell Comparison	T cell–adaptive	T cell–innate	Macrophages^*^	Fibroblasts^*^
B6 WT	+	+	1>2>6	8>13 = 21>18 = 5 = 7 = 10
RAG1 KO	-	-	6>2>1	13>8>5>10>7 = 18 = 21
B6 WT	+	+	1>2>6	8>21 = 13>18>5 = 7 = 10
OT-II	-	+	2>6>1	5>10>13>7 = 8>18>21
MyD88^fl/fl^ (Cre-)	+	+	1>2>6	8>21 = 13>18 = 5 = 7 = 10
CD4-Cre.MyD88^fl/fl^	+	-	6>2>1	13>8>18>10 = 5 = 7 = 21

*relative ranking by number of cells per cluster.

Key macrophage and fibroblast cell clusters distinguishing B6 WT mice from designated mutant strains are highlighted in red.

**Table 3B:** Macrophage and Fibroblast Subclusters.

Macrophage Clusters	Fibroblast Clusters
1-Mcemp1 F10 Fpr1 Fpr2 Mac	5-Pi16 Mfap5 Efemp1 Il33 Fibroblast
2-Marco Gbp2b Clec10a Mac	7-Cxcl14 Mme Inmt Il17d Fibroblast
6-Trem2 Spp1 Msr1 Acp5 Mac	8-Bmp3 Aldh1a3 Vegfd Fibroblast
19-Cd209f Cd209g Cd163 Lyve1 Mac	10-Gdf10 Ccn5 Dkk2 Fibroblast
22-Dcstamp Ear2 Retnla Cd226 Mac	13-Tnc Acta 2 Lrrc15 Ccn4 Adam12 Fibroblast
33-Siglec1 Marco Mertk Mac	18-Has2 Itga11 Dpp4 Fibroblast
37-Treml4 Cd300e Ear2 Mac	21-Serina3f Serpina3n Fibroblast
	36-Vit Smim41 Fibroblast
	45-Cxcl5 Col20a1 Fibroblast

**Figure 6:**
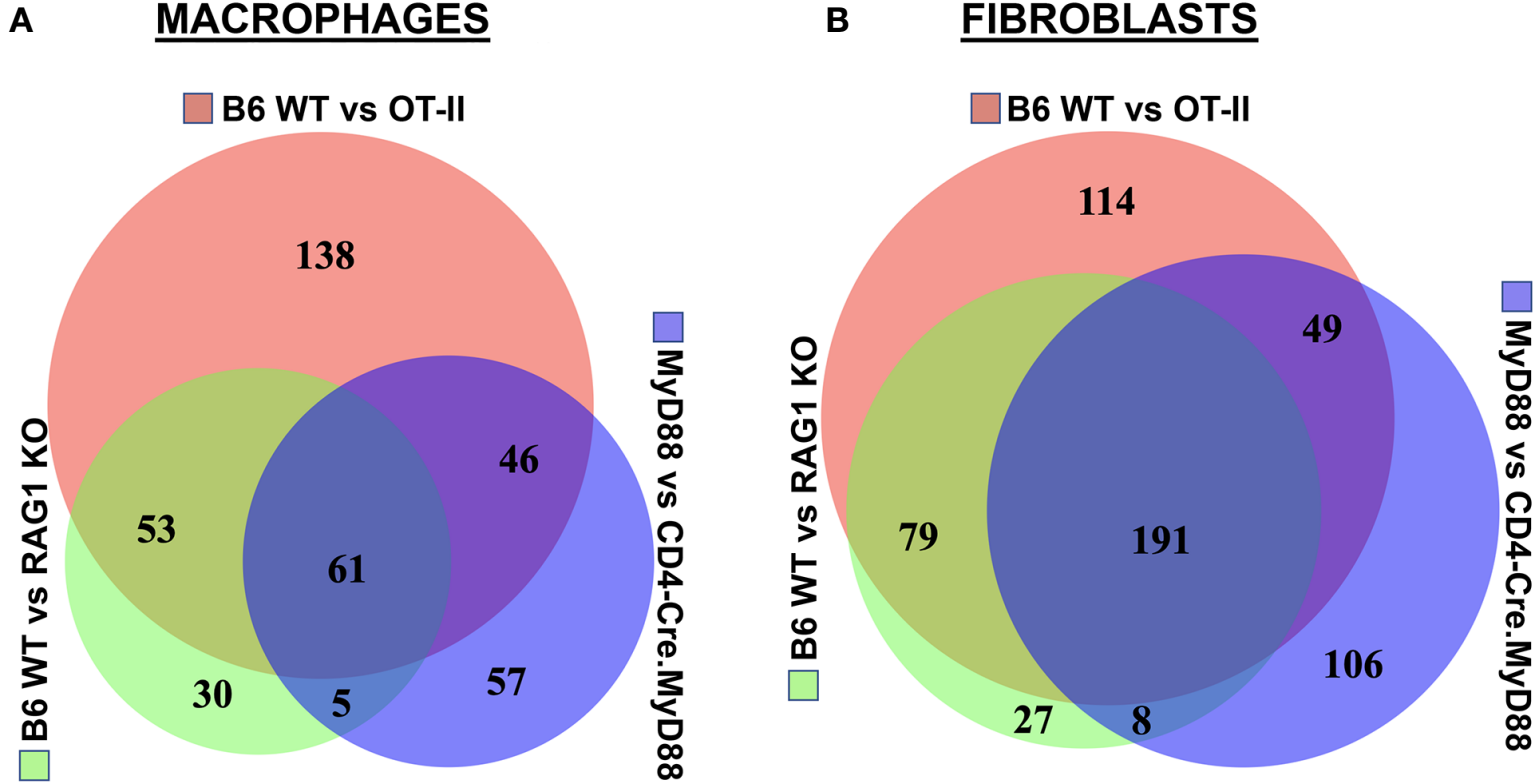
Association between T cell phenotype and differential activation of macrophage as well as fibroblast signaling pathways in HRS-induced myositis. Venn diagrams in panels **(A, B)** illustrate differential pathway induction in muscle-infiltrating macrophage and fibroblast populations (determined by Gene Ontology analysis of differentially expressed genes) following immunization of the indicated strains with recombinant HRS. Specific pathways demonstrating more than 5-fold enrichment in these inter-strain comparisons (WT vs. OT-II TCR transgenic, CD4-Cre.MyD88^fl/fl^ conditional KO, and/or RAG1 KO) are enumerated in overlapping segments of the Venn diagrams and summarized in **Supplementary Tables 3**, **4**.

Assessment of gene expression profiles corresponding to macrophage clusters 1, 2, and 6 indicate vastly different phenotypes, particularly in cluster 1 where the macrophages express a number of pro-inflammatory genes linked to α/β T cell activation, T helper cell differentiation, T cell proliferation/migration, cytokine production (IL-1β, IL-2, IL-6, IL-12, IL-17, IFNγ, TNFα, VEGF), TLR/innate immune signaling, and endothelial cell activation (**Supplementary Table 2**). This phenotype contrasts with clusters 2 and 6, in which macrophages express genes associated with wound healing, cellular chemotaxis, response to lipoproteins, cytoskeletal structural organization, lipid metabolism, iron transport, and bone remodeling (**Supplementary Table 3**). Importantly, gene expression profiles and corresponding signaling pathways within these collective macrophage subpopulations are drastically altered in OT-II TCR transgenic, CD4-Cre.MyD88^fl/fl,^ and RAG1 KO mice relative to WT mice following disease induction, as shown by the Venn diagram (18) in **Figure 6A** (which enumerates differentially expressed pathways in macrophage populations derived from these strains). Analysis of overlapping as well as divergent signaling networks (**Supplementary Table 4**) indicates that fully activated T cells drive macrophages toward a pro-inflammatory phenotype which supports antigen processing/presentation, TCR activation, T cell proliferation, cytokine production (Type 1 IFN, IFNγ, IL-1, and IL-17), and innate immune/TLR signaling—reflecting the predominance of Mcemp1-F10-Fpr1-2 macrophages populating cluster 1.

### T cell-macrophage interactions impact fibroblast phenotype

Given the extraordinary influence of T cell signaling/activation on macrophage phenotype in HRS-induced myositis and the important functional interactions between macrophages and fibroblasts in other autoimmune diseases such as scleroderma and rheumatoid arthritis (19, 20), we also assessed the transcriptional profiles of fibroblast subsets in WT, OT-II TCR transgenic, CD4-Cre.MyD88^fl/fl,^ and RAG1 KO mice following HRS immunization. Paralleling the observed shifts in macrophage subpopulations, the distribution of fibroblast subsets was significantly altered in RAG1 KO, OT-II TCR transgenic, and CD4-Cre.MyD88^fl/fl^ conditional KO mice relative to WT mice—with clear shifts in the ratios of fibroblast clusters and relative expansion of cluster 13 in knockout/transgenic mice lacking fully activated T cells (**Figure 5**). Because cluster 13 corresponds to myofibroblasts (based on expression of Tnc, Acta 2, Lrrc15, Ccn4, and Adam12 marker genes), these results indicate that a combination of innate and adaptive immune signaling pathways in activated T cells from WT mice directly or indirectly (via macrophage-derived signals) drive fibroblasts away from a pro-fibrotic phenotype mediated by myofibroblasts towards a more pro-inflammatory state. This conclusion is supported by preferential activation of pro-inflammatory signaling pathways regulating antigen presentation, T cell activation/differentiation, and cytokine/chemokine responses in WT fibroblasts (**Figure 6B** and **Supplementary Table 5**) as well as WT muscle satellite cells (**Supplementary Table 6**)—and the relative lack of muscle tissue fibrosis in WT mice at various time points post immunization (data not shown).

**Figure 7:**
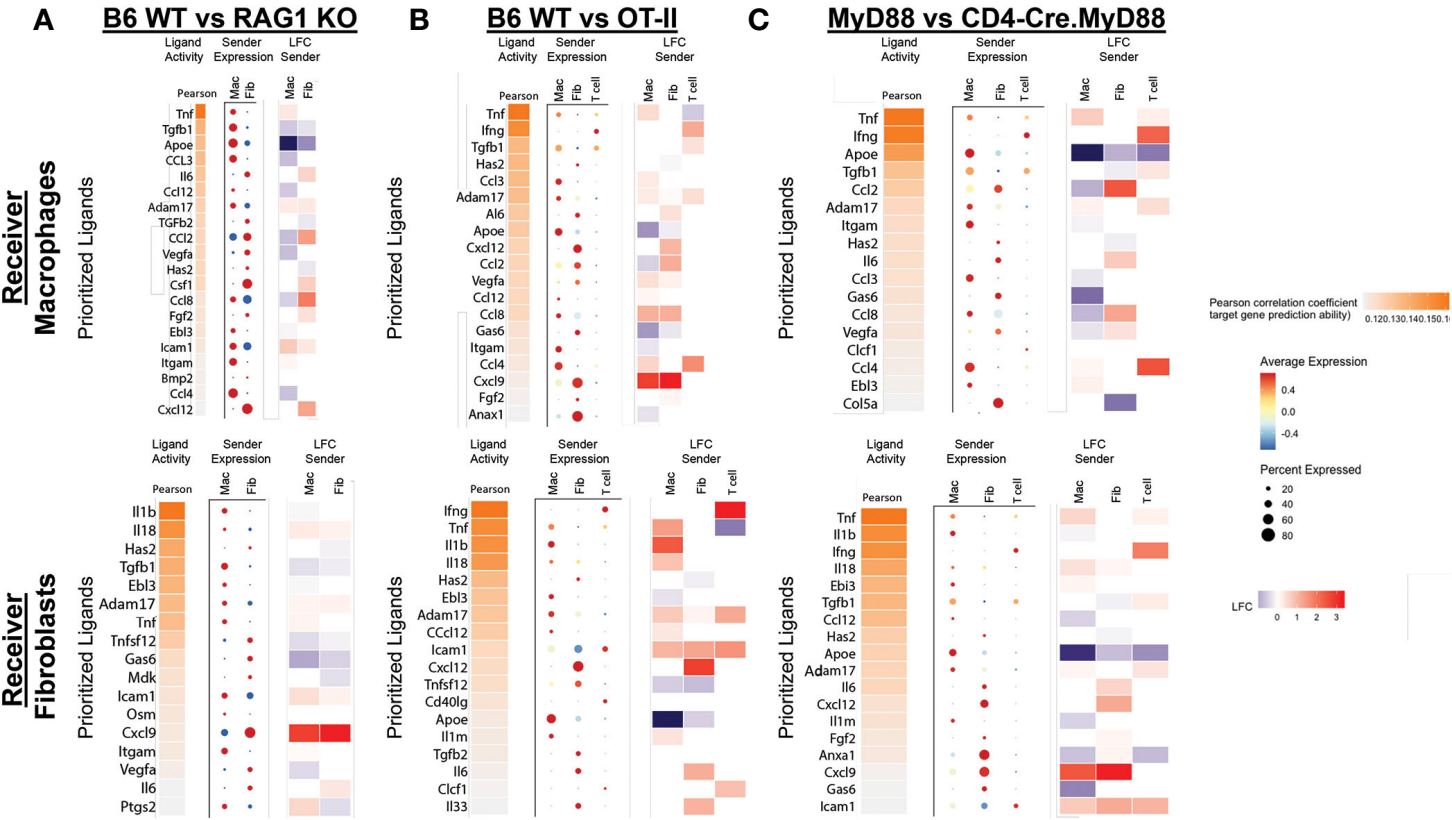
NicheNet analysis of ligand-receptor interactions in HRS-induced myositis. **(A–C)** depict predicted ligands responsible for engagement between T cells, macrophages, and fibroblasts based on differential expression of target genes in macrophages and fibroblasts. While the accompanying heat maps correspond to fold change in ligand gene expression for “sender” populations in the indicated strains (condition of interest versus reference condition), dot plots represent the relative expression level (color) and percentage of specified cells (size) expressing genes encoding these ligands. Predicted ligands are ranked based on Pearson correlation coefficients.

To further interrogate interaction networks linking T cells, macrophages, and fibroblasts, we performed NicheNet analysis (17), in which target cell gene expression profiles can be used to infer the production/secretion of specific receptor ligands. **Figure 7** indicates that multiple cell types contribute to the signaling milieu favoring activation of macrophages and fibroblasts which are responsible for promoting key inflammatory cascades underlying HRS-induced myositis. While TNFα, IFNγ, TGFβ, IL-6, CCL2, CCL3, CCL4, CXCL9, and CXCL12 drive the activation of macrophages in WT versus RAG1 KO, OT-II, and CD4-Cre.MyD88^fl/fl^ conditional KO mice, TNFα, IFNγ, IL-1β, IL-6, IL-8, CXCL9, and CXCL12 represent key cytokines/chemokines promoting pro-inflammatory pathways in WT fibroblasts. Although many of these mediators are produced by multiple cell types that include macrophages and fibroblasts themselves (suggesting significant cross-talk as well as autocrine and/or paracrine feed-forward signaling), T cells (and NK cells) are primarily responsible for elaboration of IFNγ—one of the predominant cytokines contributing to both macrophage and fibroblast activation in this model system. Collectively, these analyses demonstrate that a combination of innate and adaptive immune signals leads to activation of IFNγ-producing TH1 cells (marked by preferential expression of Tbet in muscle-infiltrating T cells of WT mice) as key drivers of the inflammatory process in HRS-induced myositis.

## Discussion

Through a series of experiments assessing the development of HRS-induced myositis in C57BL/6-derived strains lacking specific components of innate and/or adaptive immune signaling pathways, we have shown that antigen-specific TCR engagement and MyD88-directed innate immune signaling cascades are both required for maximal T cell infiltration and expression of the myositis tissue phenotype. Alterations in T cell activation have a profound functional impact on both macrophage and fibroblast subsets, as evidenced by marked skewing of gene expression profiles and phenotypic clustering of these non-lymphoid cell types in RAG1 KO, OT-II TCR transgenic, and CD4-Cre.MyD88^fl/fl^ conditional KO mice relative to WT mice. Integration of scRNAseq datasets with immunohistochemical and flow cytometric characterization of cellular infiltrates demonstrates that, in WT mice, activated T cells drive the influx of pro-inflammatory Mcemp1-F10-Fpr1-2 macrophages (cluster 1) and Bmp3-Aldh1a3-Vegfd fibroblasts (cluster 8). In contrast, mice lacking fully activated T cells demonstrate a pro-fibrotic phenotype in which Marco-Gbp2b-Clec10a (cluster 2) and Trem2-Spp1-Msr1-Acp5 (cluster 6) macrophages predominate along with Tnc-Acta 2-Lrrc15-Ccn4-Adam12 myofibroblasts (cluster 13).

More broadly, these results highlight the interdependent contributions of innate and adaptive immune responses *that are both driven by HRS* in our novel model of HRS-induced myositis. In particular, the cellular phenotype and transcriptomic profile of CD4-Cre.MyD88^fl/fl^ conditional KO mice show that disruption of MyD88-dependent signaling in T cells impacts not only antigen-driven T cell responses (consistent with findings from other investigative groups (21, 22)), but also innate immune pathways and downstream gene expression profiles in macrophages and fibroblasts. At the same time, disruption of MyD88 signaling within the myeloid cell compartment significantly impacts (but does not eliminate) T cell infiltration in Lyz2-Cre.MyD88^fl/fl^ conditional KO mice, further demonstrating the reciprocal nature of cellular interactions mediated by innate immune signaling cascades in HRS-induced myositis. Previous work has also shown the importance of coordinated MyD88 signaling in multiple cell types, as global MyD88 knockout mice yield an even more dramatic reduction in myositis than conditional knockout mice following HRS immunization (13). Conversely, the profound decrease in HRS-induced infiltration of CD45^+^ mononuclear cells (including T cells, B cells, and macrophages) in OT-II TCR transgenic mice underscores the equally significant role of adaptive immune signaling in this model—a point that is substantiated by the activation of pathways involved in antigen processing/presentation, TCR signaling, T cell differentiation, and T cell proliferation. Collectively, these data suggest that both innate and adaptive immune signaling (particularly within T cells) are necessary for full expression of the myositis phenotype, but neither alone is sufficient. This pivotal interaction between innate and adaptive immune signaling driven by HRS-mediated TLR (13) *and* TCR engagement replicates features of human IIM, in which there is ample evidence that both innate (prominence of macrophages, expression of TLRs/MyD88 signaling intermediates in muscle tissue and muscle-infiltrating cell populations) (14–16, 23–25) and adaptive (antigen-specific T cells, class-switched autoantibodies) immunity contribute to the disease process.

To further explore the immunopathogenesis of IIM, several alternative murine models have been developed over the last 20 years. In one of these models devised by Nagaraju et. al., inducible upregulation of MHC Class I promotes the influx of inflammatory cells (predominantly macrophages and T cells) 10-12 weeks post-induction of MHC Class I (26); however, biochemical alterations that predate the development of inflammatory infiltrates suggest that immune mechanisms may be playing a secondary role in this model (27). Additional models, including those based on immunization with adjuvant emulsions containing myosin or C-protein (a myosin-binding protein) (28–30), demonstrate a more direct role for (presumed) antigen-specific T cells that invade non-necrotic muscle fibers. More recently, Okiyama et al. have developed a model of dermatomyositis driven by antigen-specific CD8+ T cells targeting TIF1γ (31). While the latter models do provide support for antigen-driven processes and adaptive immunity in IIM, they do not focus on contributions from non-lymphocyte populations or fully define the role of innate immune signaling pathways.

Beyond the requirement for both innate and adaptive immune signaling in HRS-induced myositis, our findings highlight the significant role played by non-lymphoid cells that include macrophages and fibroblasts. Involvement of these non-lymphoid cell types is directly demonstrated by HRS-induced activation of macrophages and fibroblasts in RAG1 KO mice lacking mature B and T cells. Although not shown, scRNAseq-derived cell clustering and transcriptomic profiles of WT mice also demonstrate significant shifts in endothelial cell gene expression reflective of an activated phenotype. This interdependence of lymphoid and non-lymphoid muscle-infiltrating cell populations is highlighted by the NicheNet analysis of **Figure 7**, which illustrates the impact of perturbations in T cell activation and ligand production on gene expression profiles of “receiver” cell populations such as macrophages and fibroblasts. Importantly, these interactive cellular networks culminate in a TH1 driven, pro-inflammatory cascade (marked by upregulation of genes encoding the TH1-associated cytokines IFNγ, TNFα, and IL-1β) that steers the disease process away from fibrosis and defines the myositis phenotype in WT mice.

Highlighting the translational value of this model system, gene expression profiles identified in activated T cell and macrophage populations overlap with those identified from bulk RNAseq analyses of human muscle tissue impacted by IIM and the anti-synthetase syndrome (32, 33). Moreover, many of the cytokines and chemokines elaborated by muscle-infiltrating cells in this model parallel findings from human myositis muscle biopsy specimens demonstrating prominent expression/upregulation of TNFα, IL-6, IL-1β, and IFNγ as well as CXCL9/10 and CCL 2/3/4/19/21 (34–36) that collectively favor TH1-driven pathology. Given the likelihood that myocytes also actively contribute to the immunological processes driving myositis and are not simply targets of the immune response, defining pro-inflammatory signaling pathways within the muscle tissue itself (e.g., NF-κB activation resulting from engagement of TLR ligands as well as pro-inflammatory cytokines) will be critical in further elucidating the IIM disease process. In fact, detailed analysis of gene expression profiles in muscle satellite cells from HRS-immunized WT mice demonstrates activation of pro-inflammatory networks regulating Type I and Type II interferon signaling, antigen presentation, T cell-mediated cytotoxicity, monocyte chemotaxis, and other processes contributing to both innate and adaptive immune responses (**Supplementary Table 6**). As we better understand the complex molecular signaling pathways driving the pathological processes of IIM through such analyses of myocytes and other non-lymphoid cell populations, identification of newer, more selective therapeutic targets will become feasible—and ultimately lead to improvements in current treatment approaches that are highly dependent on more global immunosuppression to control this potentially devastating disease.

## Data availability statement

Single cell RNA sequencing data forming the basis of corresponding Gene Ontology Pathway analyses and NicheNet analyses encompassed by **Figures 5**, **6**, **7**, **Tables 1**, **2**, **3A**, and **Supplementary Tables 1**, **2**, **3**, **4**, **5** are available through the NIH Gene Expression Omnibus: GSE Accession number 229059.

## Ethics statement

The animal study was approved by University of Pittsburgh Institutional Care and Use Committee. The study was conducted in accordance with the local legislation and institutional requirements.

## Author contributions

Design of experiments: DR, TO, WJ, PC, and DA. Performance of experiments: DR, TT, YW, NY, and DA. Data interpretation: TT, TO, RL, WJ, PC, and DA. Drafting manuscript: DA. Editing manuscript: DR, TT, TO, NY, RL, WJ, PC, and DA. Co-first authors DR and TT made equally important contributions to this manuscript. While DR was responsible for assisting in the design/performance of *in vivo* experiments and writing of the manuscript, TT directed all of the experimental protocols and computational analysis surrounding single cell sequencing studies. The order of shared first authorship was determined by length of involvement in this project. All authors contributed to the article and approved the submitted version.
