# Supplementary material for: Antigen-driven T cell-macrophage interactions mediate the interface between innate and adaptive immunity in histidyl-tRNA synthetase-induced myositis
Source: Front Immunol. 2023 Sep 22;14:1238221. doi: 10.3389/fimmu.2023.1238221 (PMC10556668; doi:10.3389/fimmu.2023.1238221)
Supplement: Supplementary file 1 [file DataSheet_1.pdf]

Online Supplemental Material. While **Supplementary Figure 1** demonstrates the flow cytometric profile of muscle-infiltrating mononuclear cells in wild type HRS-induced myositis, **Supplementary Figures 2-4** illustrate the distribution and intensity of inflammatory infiltrates in muscle tissue of CD4 KO, CD4-Cre.MyD88$^{fl/fl}$ conditional KO, and IL-1R KO relative to WT mice follow HRS-mediated induction of myositis. **Supplementary Figure 5** shows comparative flow cytometry profiles and a tabular summary of muscle-infiltrating cell populations in CD4-Cre.MyD88$^{fl/fl}$ conditional KO versus MyD88$^{fl/fl}$ littermate control mice. Based on scRNAseq-derived clustering algorithms, **Supplementary Table 1** shows the distribution of muscle-resident and muscle-infiltrating cells in different congenic strains of C57BL/6 mice following immunization with PBS versus recombinant HRS. **Supplementary Table 2** catalogues Gene Ontology-derived pathways preferentially activated in Mcemp1-F10-Fpr1-2 macrophages (cluster 1) that are over-represented in WT mice following HRS-mediated induction of myositis. **Supplementary Table 3** enumerates pathways activated in Marco-Gbp2b-Clec10a (cluster 2) and Trem2-Spp1-Msr1-Acp5 (cluster 6) macrophages derived from WT mice immunized with recombinant HRS. **Supplementary Tables 4-5** list Gene Ontology-derived pathways that distinguish different macrophage (**Supplementary Table 4**) and fibroblast (**Supplementary Table 5**) populations in WT, RAG1 KO, CD4-Cre.MyD88$^{fl/fl}$ conditional KO, and OT-II TCR transgenic mice following induction of myositis. Finally, **Supplementary Table 6** catalogues HRS-induced pro-inflammatory signaling pathways in Pax7 muscle satellite cells isolated from WT mice.

SUPPLEMENTARY FIGURE LEGENDS

Supplementary Figure 1. Profile of muscle-infiltrating mononuclear cells in HRS-induced myositis. Dot plots illustrate flow cytometric profiles of muscle-infiltrating cell populations derived from B6 WT mice following HRS-mediated induction of myositis. Cell surface markers of T cells (CD3), B cells (CD19), NK cells (CD161), and macrophages (F4-80) are indicated on the x-axes. $CD4^+$ T cells were quantified as a percentage of $CD45^+CD3^+CD90.2^+$ cells, whereas CD19+ B cells, $CD161^+$ NK cells and $F4\text{-}80^+$ macrophages were gated from $CD45^+CD3^-$ cell populations.

Supplementary Figure 2. Requirement for $CD4^+$ T cells in HRS-induced myositis. Panel **(A)** demonstrates H&E- and immunohistochemically-stained muscle tissue derived from n=5 CD4 KO and n=5 B6 WT mice 17 days post immunization with recombinant HRS. Cell surface markers of infiltrating T cells (CD3, CD4, CD8) and macrophages (CD68) are indicated in the upper right hand corner of individual tissue sections. Scale bars (400 μm) are shown in the lower right hand corner of each photomicrograph. Box plots in panel **(B)** demonstrate the relative severity of muscle inflammation/cellular infiltration in CD4 KO versus B6 WT mice following immunization with recombinant HRS, with horizontal bars representing median scores an "x" symbols designating mean levels of inflammation; median (IQR)=1.5 (1.5, 2) CD4 KO vs. 3 (2.5, 3) B6 WT; mean=1.7 CD4 KO vs. 2.8 B6 WT; min/max=1.5/2.5 CD4 KO vs. 2.5/3 B6 WT; p-value determined by Mann-Whitney U-test.

Supplementary Figure 3. Muscle-infiltrating T cell profile in C57BL/6 WT versus CD4-Cre.$MyD88^{fl/fl}$ conditional knockout mice. While panel **(A)** demonstrates cell surface expression of CD3, CD4, and CD8 in muscle-infiltrating lymphocytes of B6 WT mice following immunization with recombinant HRS, panel **(B)** shows corresponding immunohistochemical staining of inflammatory infiltrates resulting from HRS immunization of CD4-Cre.$MyD88^{fl/fl}$ conditional knockout mice.

Supplementary Figure 4. HRS-induced myositis in IL-1 Receptor KO mice. Panel **(A)** demonstrates low (40X) and high (100x) powered magnification of H&E-stained muscle tissue from IL-1R KO and C57BL/6 (B6) WT mice 17 days post immunization with recombinant HRS. Scale bars (1000 μm for low power and 400 μm for high power images) are shown in the lower right hand corner of each photomicrograph. Box plots in panel **(B)** illustrate the relative severity of muscle inflammation/cellular infiltration in n=5 IL-1R KO versus n=5 B6 WT mice following immunization with recombinant HRS. Median and mean severity scores are indicated by horizontal bars and "x" symbols, respectively; median (IQR)=3 (2.25, 3) IL-1R KO vs. 3 (2.5, 3) B6 WT; mean=2.7 IL-1R KO vs. 2.8 B6 WT; min/max=2/3 IL-1R KO vs. 2.5/3 B6 WT; p-value determined by Mann-Whitney U-test.

Supplementary Figure 5. Differential profile of muscle-infiltrating mononuclear cells in $MyD88^{fl/fl}$ versus CD4-Cre.$MyD88^{fl/fl}$ mice. Dot plots illustrate flow cytometric profiles of muscle-infiltrating cell populations derived from $MyD88^{fl/fl}$ versus CD4-Cre.$MyD88^{fl/fl}$ mice following immunization with recombinant HRS. Cell surface markers of T cells (CD3), B cells (CD19), NK cells (CD161), and macrophages (F4-80) are indicated on the x-axes. While $CD19^+$ B cells were gated as a percentage of $CD45^+CD3^-$ populations, $CD161^+$ NK cells and $F4\text{-}80^+$ macrophages were quantified from $CD3^-CD19^-$ and $CD3^-CD19^-CD161^-$ cells, respectively. Numbers of viable CD3+ T cells, CD19+ B cells, CD161+ NK cells, and F4-80+ macrophages are summarized in the accompanying table; given the similar number of live cells isolated from muscle for each experimental condition, percentages of cell subsets relative to number of live cells have been omitted for ease of illustration.
