# Supplementary figures and images for: Antigen-driven T cell-macrophage interactions mediate the interface between innate and adaptive immunity in histidyl-tRNA synthetase-induced myositis
Source: Front Immunol. 2023 Sep 22;14:1238221. doi: 10.3389/fimmu.2023.1238221 (PMC10556668; doi:10.3389/fimmu.2023.1238221)

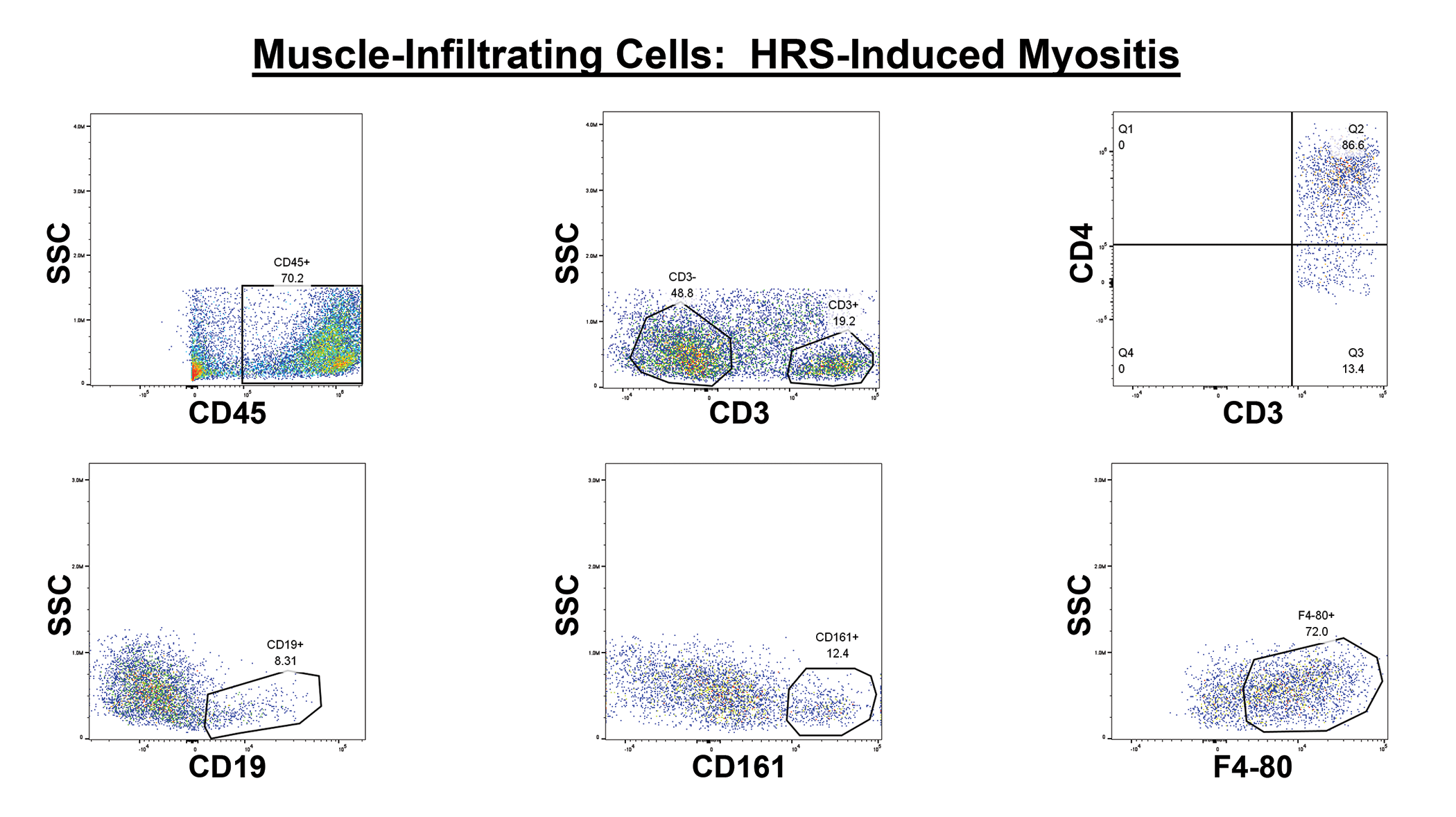

Supplement: Supplementary file 2 [file Image_1.tif]

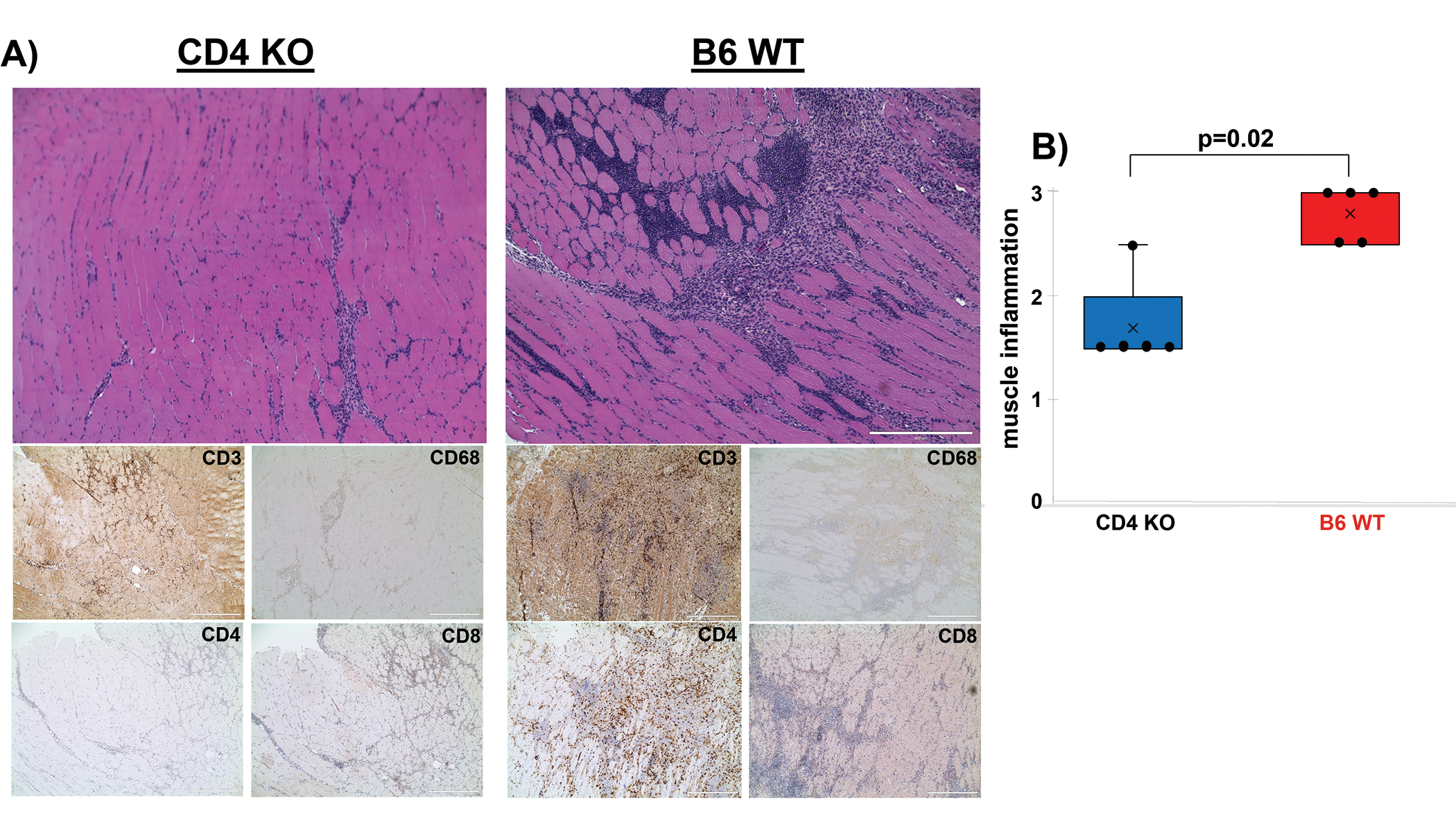

Supplement: Supplementary file 3 [file Image_2.tif]

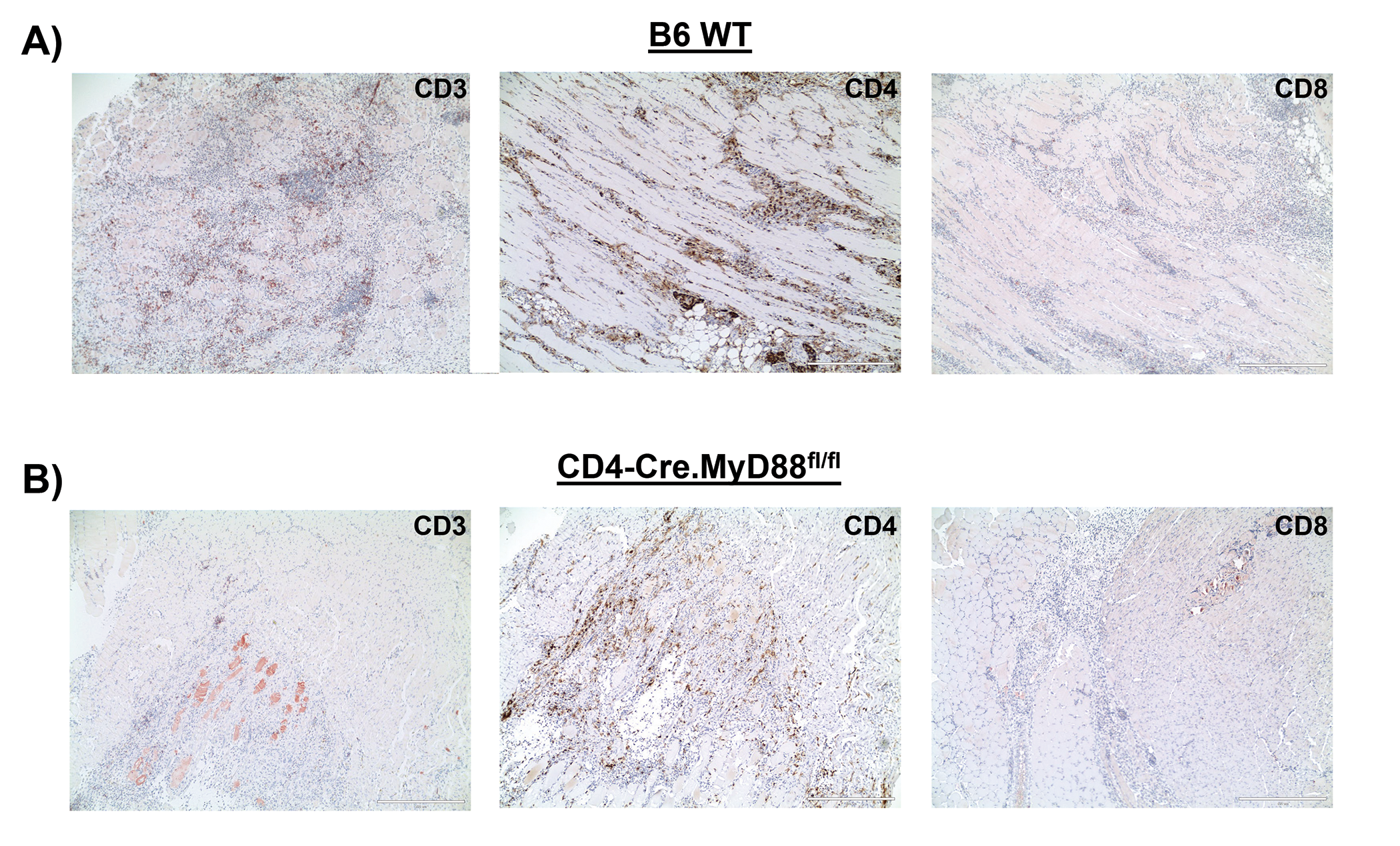

Supplement: Supplementary file 4 [file Image_3.tif]

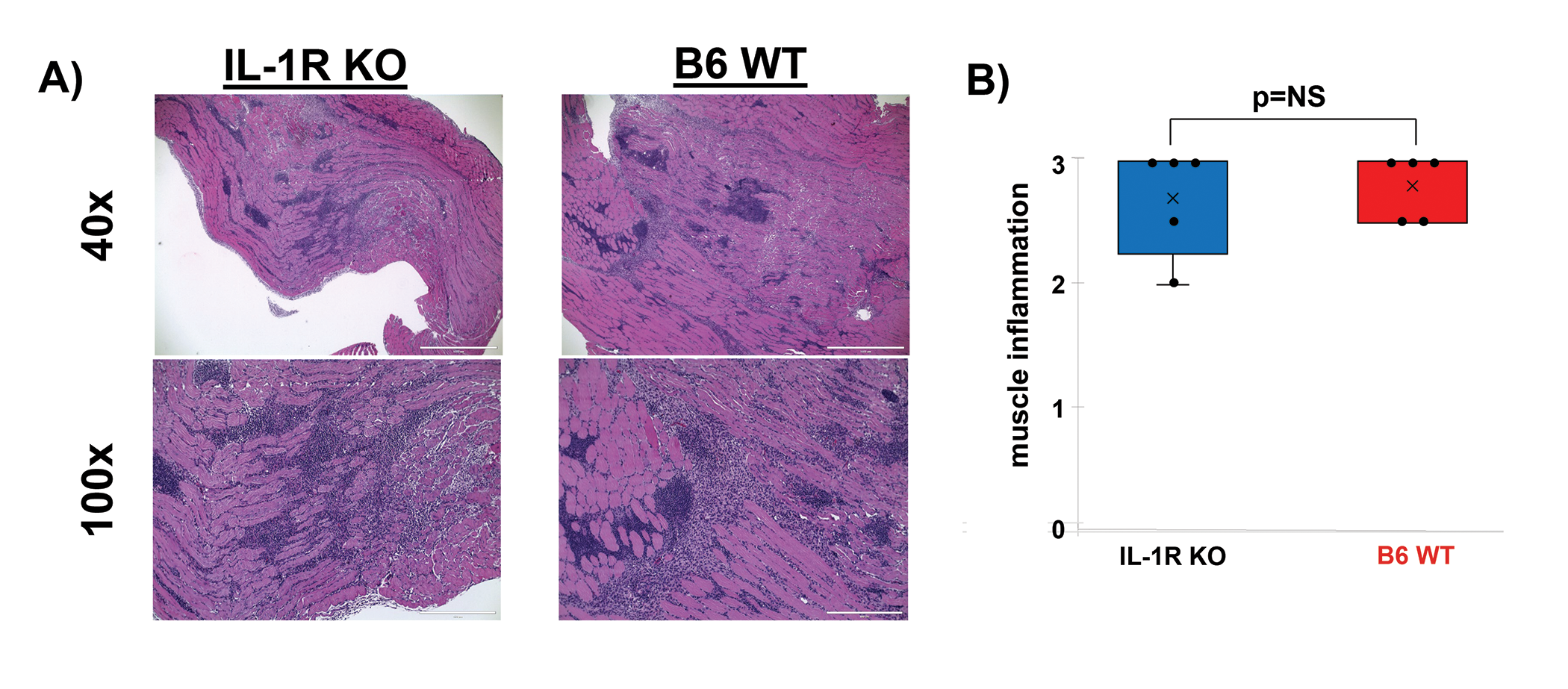

Supplement: Supplementary file 5 [file Image_4.tif]

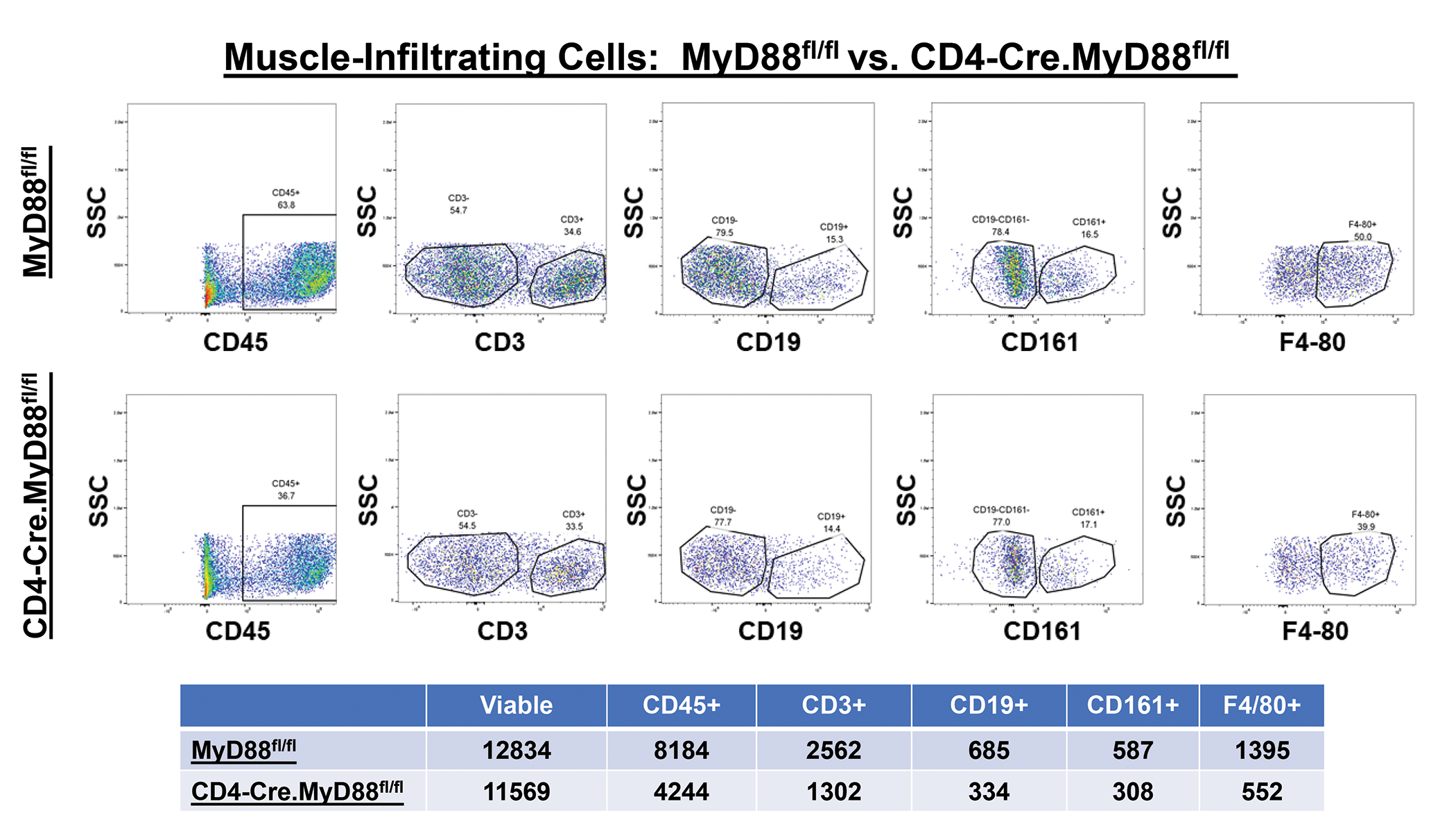

Supplement: Supplementary file 6 [file Image_5.tif]
